# The AhR agonist VAF347 augments retinoic acid-induced differentiation in leukemia cells

**DOI:** 10.1016/j.fob.2015.04.002

**Published:** 2015-04-08

**Authors:** Christopher N. Ibabao, Rodica P. Bunaciu, Deanna M.W. Schaefer, Andrew Yen

**Affiliations:** a Department of Biomedical Sciences, Cornell University, Ithaca, NY 14853, USA; b Department of Population Medicine and Diagnostic Sciences, Cornell University, Ithaca, NY 14853, USA

**Keywords:** AhR, aryl hydrocarbon receptor, APC, allophycocyanin, APL, acute promyelocytic leukemia, D_3_, 1,25-dihydroxyvitamin D3, FICZ, 6-formylindolo (3,2-b) carbazole, GM, granulo-monocytic, PE, phycoerythrin, RA, all *trans*-retinoic acid, TCDD, 2,3,7,8-tetrachlorodibenzo-p-dioxin, VAF347, (4-(3-Chloro-phenyl)-pyrimidin-2-yl)-(4-trifluoromethyl-phenyl)-amine, Retinoic acid, Differentiation, Leukemia, VAF347

## Abstract

In binary cell-fate decisions, driving one lineage and suppressing the other are conjoined. We have previously reported that aryl hydrocarbon receptor (AhR) promotes retinoic acid (RA)-induced granulocytic differentiation of lineage bipotent HL-60 myeloblastic leukemia cells. VAF347, an AhR agonist, impairs the development of CD14^+^CD11b^+^ monocytes from granulo-monocytic (GM) stage precursors. We thus hypothesized that VAF347 propels RA-induced granulocytic differentiation and impairs D_3_-induced monocytic differentiation of HL-60 cells. Our results show that VAF347 enhanced RA-induced cell cycle arrest, CD11b integrin expression and neutrophil respiratory burst. Granulocytic differentiation is known to be driven by MAPK signaling events regulated by Fgr and Lyn Src-family kinases, the CD38 cell membrane receptor, the Vav1 GEF, the c-Cbl adaptor, as well as AhR, all of which are embodied in a putative signalsome. We found that the VAF347 AhR ligand regulates the signalsome. VAF347 augments RA-induced expression of AhR, Lyn, Vav1, and c-Cbl as well as p47^phox^. Several interactions of partners in the signalsome appear to be enhanced: Fgr interaction with c-Cbl, CD38, and with pS259c-Raf and AhR interaction with c-Cbl and Lyn. Thus, we report that, while VAF347 impedes monocytic differentiation induced by 1,25-dihydroxyvitamin D_3_, VAF347 promotes RA-induced differentiation. This effect seems to involve but not to be limited to Lyn, Vav1, c-Cbl, AhR, and Fgr.

## Highlights

• VAF347 augments RA-induced expression of Vav1, c-Cbl, Lyn, and P47^phox^.
• VAF347 augments RA-induced G0/G1 enrichment.
• VAF347 augments RA-induced expression of CD11b integrin receptor.
• VAF347 augments RA-induced respiratory burst activity of neutrophils.

## Graphical abstract

**Figure:**
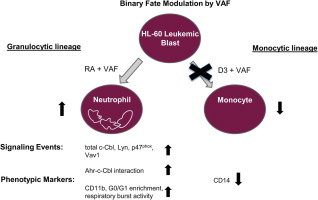

## 1 Introduction

Acute promyelocytic leukemia (APL) is a distinct form of acute myeloid leukemia (AML) that is characterized by an arrest of leukocyte differentiation at the promyelocyte stage. Standard treatment of APL to mitigate the block includes differentiation therapy with all *trans*-retinoic acid (RA) [1]. However, despite advances in differentiation induction therapy and purported complete remission rates of 80–85%, some APL patients develop either relapsed or refractory disease which no longer responds to RA therapy. Therefore, insights into novel pathways and molecular agents that enhance leukemic cell differentiation are sought to render RA efficacious where it is presently not.

RA is a fundamental regulator of cell proliferation and differentiation. It guides embryogenesis and controls differentiation in processes such as immune response in mature animals, as well. RA’s mechanism of action is thus of basic biological interest as well as for therapy. Insights into how it elicits responses in non-APL cells is thus of potential significance to enhancing therapy. Aryl hydrocarbon receptor (AhR) is a multifaceted signaling transducer. As a transcription factor, AhR is known to be involved in tumorigenesis and proliferation of several stomach, breast, and liver cancers; further, it has also been found to be implicated in cecal carcinogenesis and even hematopoietic stem cell growth [2,3]. AhR is also known to have several anti-tumorigenic effects and homeostatic regulatory roles within the body, and it does so in a ligand agonist- or antagonist-specific manner [2,4,5]. In this regard, AhR can regulate tumor cell differentiation to a non-tumor phenotype. However, it is not yet clear whether AhR regulates such differentiation solely through its classical transcriptional activity. In the case of retinoic acid (RA)-induced granulocytic differentiation of human myeloblastic leukemia cells, we reported that AhR promotes the RA-induced differentiation of myeloblastic leukemia cells by restricting the nuclear expression of stem cell transcription factor Oct4 [6]. Furthermore, other studies have reported AhR as a component of an ubiquitin ligase complex involved in regulating the degradation of select target proteins [7]. We also found that AhR can drive cytoplasmic signaling via the Raf kinase of the MAPK pathways to propel RA-induced differentiation of the leukemic cells [6]. Hence AhR may regulate tumor cells through both its classical nuclear transcriptional role as well as cytoplasmic non-transcriptional mechanisms.

**Fig. 1:**
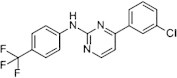
VAF347 structural formula. (4-(3-Chloro-phenyl)-pyrimidin-2-yl)-(4-trifluoromethyl-phenyl)-amine.

AhR activity is regulated by ligands. It has been reported that AhR’s transcriptional activity may be differentially elicited by exogenous ligands such as 2,3,7,8-tetrachlorodibenzo-p-dioxin (TCDD), or endogenous ligands such as the tryptophan metabolite 6-formylindolo (3,2-b) carbazole (FICZ) [8]. We previously reported that FICZ also enhances RA-induced signaling events and propels leukemic cell differentiation [9]. Therefore, AhR and its ligands are attractive therapeutic targets for enhancing the induction of leukemic cell differentiation [2], and mechanistic insights of ligated AhR-regulated processes are likely to assist drug development efforts. To this end, the effects of an AhR agonist known as VAF347, i.e., (4-(3-Chloro-phenyl)-pyrimidin-2-yl)-(4-trifluoromethyl-phenyl)-amine (Fig. 1), on signaling and RA-induced cell differentiation and cell cycle arrest was analyzed *in vitro*. VAF347 is a low molecular weight, cell permeable compound that directly targets AhR with great affinity and induces downstream AhR signaling. This relatively novel compound causes a number of anti-inflammatory responses *in vitro* and *in vivo* [10]. It inhibits the development of CD14^+^CD11b^+^ monocytes from granulo-monocytic (GM stage) precursors [11]. There is reason to suspect that VAF347 may enhance the anti-cancer effects of RA. Specifically, leukemic blasts that are differentiation-blocked at the granulo-monocytic (GM) stage differentiate into neutrophils upon RA exposure; whereas GM-precursors treated with 1,25-dihydroxyvitamin D3 (D_3_) differentiate along the alternative monocytic lineage. VAF347 has been found to inhibit monopoiesis [11]. If driving one lineage and suppressing the other are conjoined in binary fate decisions [12,13] then VAF347 might in fact propel RA-induced granulocytic differentiation.

In the present study, the effect of VAF347 on RA-induced differentiation and cell cycle arrest and the signaling events thought to propel this were determined. It is known that the phenotypic conversion elicited by RA is driven by an ensemble of signaling molecules assembled in a signalsome [14–16]. The putative signalsome incorporates in particular CD38 anchored c-Cbl and its associated signaling partners, including c-Raf, Fgr, Lyn, Vav1 as well as AhR. We demonstrate that VAF347 regulates Lyn, Vav1, and c-Cbl, components of the signalsome, and enhances RA-induced differentiation along the granulocytic lineage.

## 2 Results and discussion

### 2.1 VAF347 retards monocytic but enhances granulocytic differentiation

**Fig. 2:**
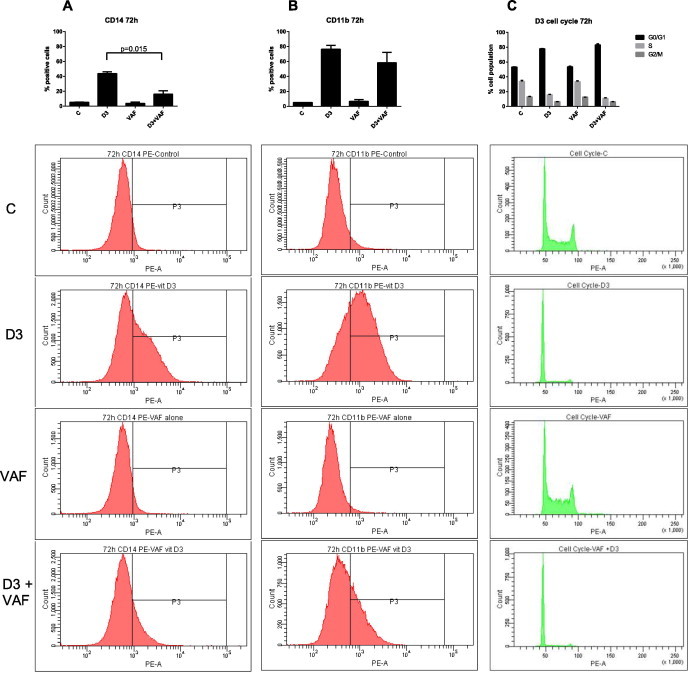
VAF347 inhibits D_3_-induced monocytic differentiation. HL-60 cells were initiated in culture with 0.5 μM D_3_, 20 μM VAF347 or both D_3_ plus VAF347 for 72 h when their differentiation was analyzed. VAF347 in combination with D_3_ blunts the expression of (A) CD14 and (B) CD11b, and enhances (C) Cell cycle arrest at G0/G1. Percent increase of expression of markers by D_3_ and/or VAF347 was set to exclude 95% of the control population for CD14 and CD11b. Means of treatment groups of interest were compared using the Paired-Samples *T* Test. Data represent the means of three repeats ± S.E.M. A *p*-value of <0.05 was considered significant.

VAF347 is an AhR agonist reported to hinder the development of CD14^+^CD11b^+^ monocytes from precursors [11]. Based on the paradigm that lineage bipotent cells excluded from one pathway have enhanced differentiation along the alternative pathway, we hypothesized that VAF347 might augment development of granulocytes from GM-precursors. Specifically, we tested the hypothesis that VAF347 enhances the RA-induced differentiation of HL-60 myeloblastic leukemia cells. First, the effect of VAF347 on 1,25-dihydroxyvitamin D_3_ (D_3_)-induced monocytic differentiation in HL-60 was tested to assess if this model is concordant with the *in vivo* mouse model where VAF347 inhibits differentiation to monocytes. Flow cytometry analysis showed that administering VAF347 concurrently with D_3_ significantly inhibits the expression of the monocytic differentiation antigen CD14, *p* < 0.02, (Fig. 2A) compared to D_3_ alone, whereas expression of the monocytic-granulocytic integrin CD11b was not significantly decreased (Fig. 2B). VAF347 together with D_3_ does not significantly augment the G0/G1 arrest (Fig. 2C). VAF347 alone had no effect on any of these three markers: CD14, CD11b or cell cycle arrest (Fig. 2).

**Fig. 3:**
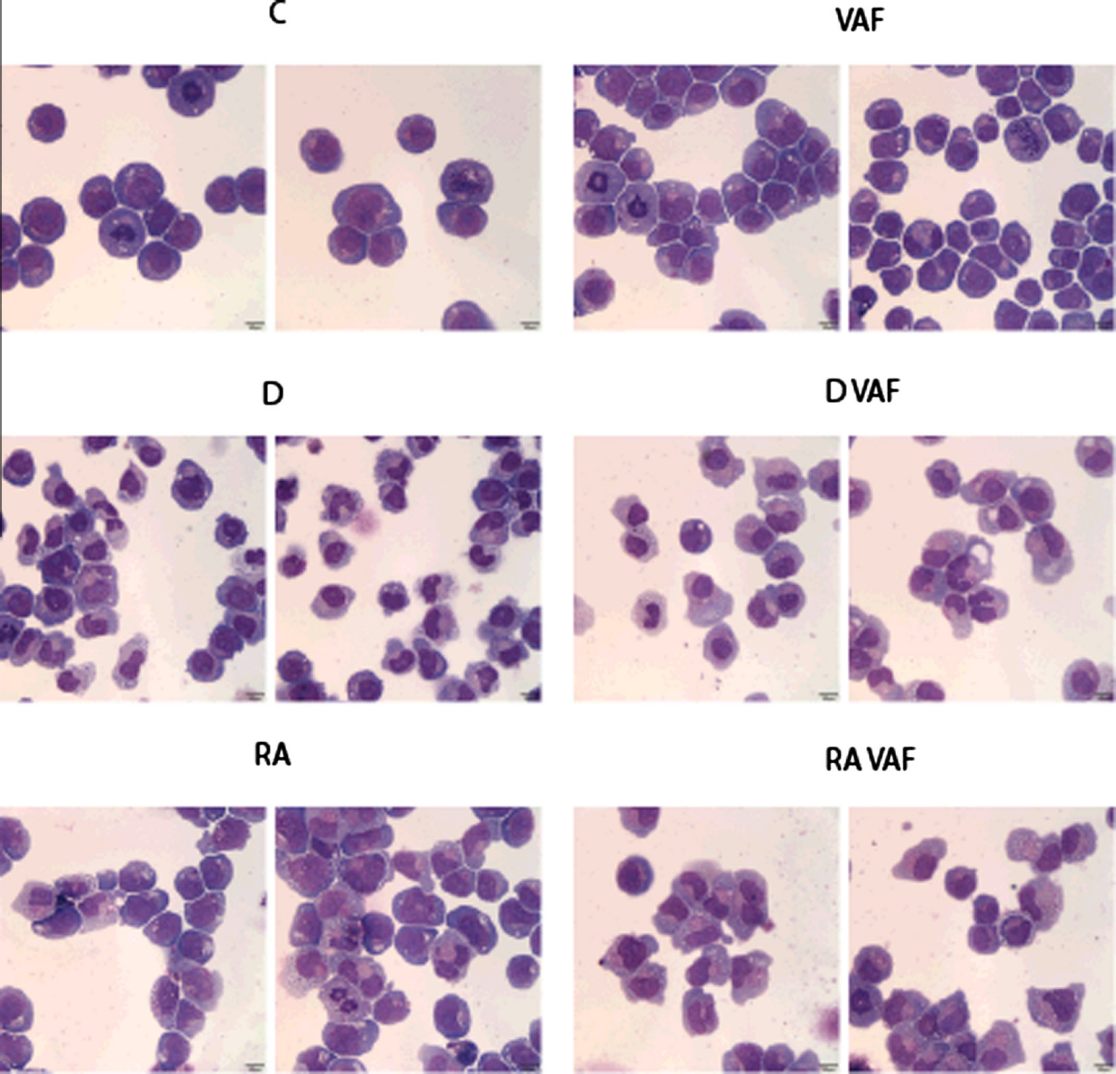
VAF347 promotes granulocytic morphology. HL-60 cells were initiated in culture with 20 μM VAF347 in combination with either 1 μM RA or 0.5 μM vitamin D_3_ for 96 h when cytocentrifuge samples were prepared and stained. C = untreated control cells; VAF = treated only with VAF347; D = treated only with vitamin D_3_; D VAF = treated with vitamin D_3_ + VAF347; RA = treated only with retinoic acid; RA VAF = treated with RA + VAF347. C and VAF are composed of undifferentiated blast cells. The remaining treatments induced variable levels of morphologically detectable granulocytic or monocytic differentiation, which are most easily detectable in the D VAF and RA VAF treatments. Wright’s stain viewed with 500× magnification.

Wright’s stain was used to assess the morphology of untreated, VAF347 only, RA only, D_3_ only, VAF347 + RA, and VAF347 + D_3_ treated cells. As shown in Fig. 3, untreated HL-60 control and cells treated only with VAF347 consisted of undifferentiated blast cells with frequent mitotic figures (Fig. 3, C and VAF). The remaining treatments induced variable levels of morphologically detectable granulocytic or monocytic differentiation. None of these treatments induced complete maturation to a segmented neutrophil, and cells often retained nucleoli, which is typically a feature of immature cells. However, HL-60 cells concurrently treated with vitamin D_3_ and VAF347 and those concurrently treated with RA and VAF347 (Fig. 3, D_3_ VAF and RA VAF) had differentiation detected in most cells indicated by an indented or lobulated nucleus and a blue-gray to pink cytoplasm color. Determination of whether this differentiation was toward the granulocytic versus monocytic lineage is difficult based solely on morphology, but, interestingly, the treatment with vitamin D_3_ plus VAF347 induced development of cells with the most convincing granulocytic differentiation, i.e., cells with clear to light pink cytoplasm. Cells treated with these combinations also had very few undifferentiated blast cells and only rare mitotic figures compared to other single agent treatments. The morphological assessment agrees with the flow cytometric measurements in that VAF347 in combination with D_3_ does allow initiation of the differentiation, but deters it from the monocytic lineage.

**Fig. 4:**
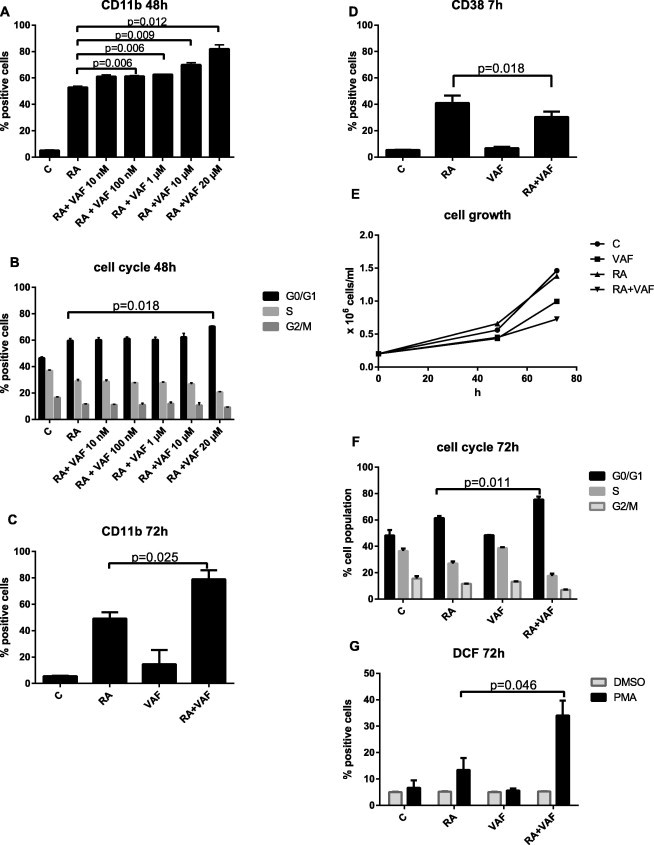
VAF347 with RA enhances expression of differentiation marker CD11b, increases G0/G1 cell cycle arrest, and functional differentiation marker oxidative metabolism (respiratory burst). HL-60 cells were exposed to either 1 μM RA or 1 μM RA with VAF347 at 10 nM, 100 nM, 1 μM, 10 μM, or 20 μM for the dose response studies at 48 h. Cells were exposed to 1 μM RA, 20 μM of VAF347 alone, or both agents for further studies. Induced differentiation and cell cycle arrest were measured at the indicated times: 7, 48, and 72 h. CD11b expression (as determined from flow cytometry with a PE-conjugated antibody) was increased by combining both RA and VAF347 as opposed to RA or VAF alone at: (A) 48 h and (C) 72 h (D) CD38 RA-induced expression (assayed with an APC-conjugated antibody) was decreased by VAF347. Gates were set to exclude 95% of the control population to determine the percent increase in expression for tested cell surface differentiation markers. (E) Cell density was measured for treated and untreated HL-60 cells at 0, 48, and 72 h. Cell growth showed that VAF347 was not toxic to the cells. G0/G1 cell cycle arrest is increased by co-administration of VAF347 and RA, compared to RA alone. Cell cycle distribution of HL-60 cells exposed to RA, VAF347 or both agents was analyzed using propidium iodide stained nuclei. Percent of cells in G0/G1, S, and G2 is shown. An increase of cells in G0/G1 and corresponding decrease in S and G2 compared to RA alone was observed at (B) 48 h and (F) 72 h. Respiratory burst is augmented at 72 h by the combination treatment of RA with VAF347 compared to RA alone. Respiratory burst was quantified using flow cytometry of dichlorofluorescein (DCF)-stained cells. VAF347 with RA increased superoxide metabolites from inducible oxidative metabolism (*p* = 0.046) at (G). Gates were set to exclude 95% of DMSO population to determine level of increased expression by VAF347. Statistical analyses were performed using GraphPad. Means of treatment groups of interest were compared using the Paired-Samples *T* Test. Data represent the means of three repeats ± S.E.M. A *p*-value of <0.05 was considered significant.

HL-60 bipotent cells treated with RA differentiate along the granulocyte lineage. In order to test whether or not VAF347 enhances this RA-induced differentiation toward the granulocytic lineage and to determine the minimal concentration of VAF347 needed to elicit such a response, a dose response study was performed at 48 h (Fig. 4A). Enhancement of RA-induced differentiation was assessed by enhancement of two markers indicative of RA-induced granulocytic differentiation: CD11b and cell cycle arrest. Thus, cells were treated with either RA or RA with VAF347 at increasing concentrations of VAF347. We found that expression of the CD11b membrane receptor (as determined from flow cytometry with a PE-conjugated antibody) was enhanced when cells were co-treated with both RA and VAF347 at 100 nM compared to RA alone (61% versus 53%, *p* = 0.006, after 48 h). Furthermore, expression of the membrane receptor increased in a dose dependent manner beyond 1 μM of VAF347 co-treatment with RA.

Enrichment at G0/G1 was also examined for at 48 h (Fig. 4B). Interestingly, G0/G1 was not enhanced at any combinatorial doses under VAF347 at 20 μM co-treatment with RA. VAF347 at 20 μM plus RA enhanced enrichment of G0/G1 compared to RA alone (70.3% versus 60%, *p* = 0.01, after 48 h). Resultantly, we found that RA with VAF347 at 20 μM when compared to RA alone was the only treatment group that was significant in enhancing both CD11b expression and cell cycle arrest at G0/G1 at 48 h. Thus, we chose VAF347 at 20 μM as the concentration of interest for further study in HL-60.

Induced expression of CD11b was subsequently measured at 72 h (Fig. 4C) of treatment. RA-induced CD11b expression was lower than that induced by co-treatment with RA and VAF347 (50% versus 74%, *p* = 0.025, after 72 h). However, as determined by flow cytometry, combined RA- and VAF347-treated HL-60 cells did not exhibit an increased cell surface expression of CD38 (Fig. 4D), an additional marker of RA-induced granulocytic differentiation, compared to RA alone. CD38 levels remained unchanged with VAF347 alone. The results found here are consistent with previous work with an endogenous AhR ligand, 6-formylindolo (3,2-b) carbazole (FICZ) in myeloblastic leukemic cells [9]. Here we find that cells treated with VAF347 with RA displayed increased expression of cell surface marker CD11b. However, CD38 expression at 7 h post treatment is decreased (*p* = 0.018). Since CD38 is one of the earliest markers of progressive differentiation and CD11b is a later marker for a more differentiated cell, differentiation was enhanced by VAF347, a suggestion corroborated by growth arrest and RA-induced functional differentiation betrayed by inducible oxidative metabolism, as shown below.

Cell densities revealed the most pronounced growth retardation due to co-treatment with RA and VAF347 (Fig. 4E). Corroborating this, cell cycle arrest betrayed by enrichment of G0/G1 cells was also enhanced by combined exposure to both RA and VAF347 (Fig. 4B, 48 h and Fig. 4F, 72 h). RA alone caused an increase in the percentage of G0/G1 cells, and co-treatment further enhanced cell cycle arrest (62% versus 78%, *p* = 0.011 after 72 h). Corresponding reductions in S phase and G2 cells were also observed. VAF347 thus enhanced RA-induced cell cycle arrest in G1/0.

**Fig. 5:**
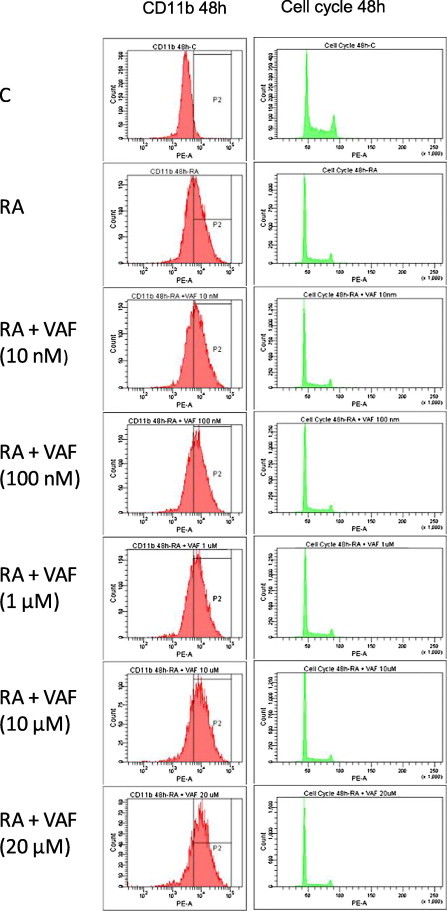
Dose response assay representative histograms. Representative histograms for CD11b PE and cell cycle for the studies quantified in Fig. 4A and B.

Enhanced induction of differentiation of human myeloblastic leukemia cells by VAF347 is also shown by respiratory burst (inducible oxidative metabolism), a functional marker of mature myeloid cells. Oxidative metabolism, a measure of the ability of neutrophils and macrophages to respond to bacterial infection, is a functional differentiation marker of mature granulocytes and monocytes. Accordingly, the ability of cells to undergo respiratory burst was measured in RA and VAF347 treated cells. Respiratory burst measured after treatment for 72 h was increased in cells treated with a combination of RA plus VAF347 compared to RA alone (34% versus 13.4%, *p* = 0.046) (Fig. 4G). However at 48 h this difference was not yet significant. In sum, VAF347 enhanced RA-induced cell cycle arrest and myeloid differentiation. The representative histograms for the assay reported in Fig. 4 are presented in Figs. 5 (dose response) and 6 (phenotypic markers).

### 2.2 Modulation of signaling events driving differentiation by VAF347

RA-induced GM-precursor cell differentiation is driven by a CD38 receptor anchored signalsome of MAPK signaling molecules; ergo we next investigated if VAF347 augments RA-induced differentiation via enhancement of the putative signalsome known to be involved in directing the differentiation process in HL-60 cells. Important components of this signalsome that are upregulated and/or activated following RA exposure include AhR, several components of the MAPK signaling cascade, Src-family kinases Lyn and Fgr, and c-Cbl. The addition of VAF347 with RA augmented the effects in particular on c-Raf, pS259c-Raf, (but notably not so for MEK or ERK), on Vav1, and c-Cbl.

**Fig. 7:**
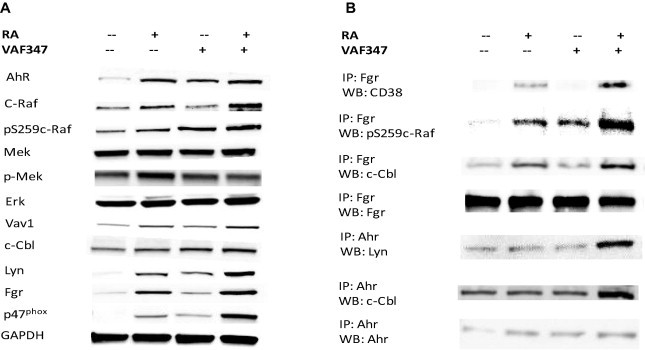
VAF347 modulates RA elicited signalsome. HL-60 cells were cultured with 1 μM RA, 20 μM VAF347, or both agents. Whole cell lysates were collected after 48 h. Lysates were resolved on a 12% polyacrylamide gel. 25 μg of protein was loaded in each well. GAPDH was used as the loading control for the western blot. (A) Western blot assay of whole cell lysates. (B) Co-immunoprecipitation representative blots. Fgr or AhR associated complexes were immunoprecipitated from protein lysates of control, RA alone, VAF347 alone, or both agents, using Fgr or AhR antibodies mentioned in Section 3. After the gel separation and membrane transfer, the co-immunoprecipitation was probed for CD38, pS259c-Raf, or c-Cbl. AhR associations were tested with Lyn or c-Cbl antibodies. Fgr and AhR immunoprecipitations served as controls for Fgr and AhR total amount pulled-down. Western blots and immunoprecipitation results are representative of at least three repeats.

Western blot analysis showed (Fig. 7A) that RA induced increased AhR levels compared to control, which correlates with differentiation as reported in previous studies [9,17]. However, adding VAF347 did not enhance this effect (Fig. 7A). RA alone, VAF347 alone, and RA with VAF347 increased AhR expression to a comparable extent. These findings are consistent with the notion that enhanced effects attributed to VAF347 do not reflect enhanced AhR expression but rather AhR’s activation.

Total c-Raf expression and its phosphorylation at pS259c-Raf were increased by combination treatment, where addition of VAF347 augments the increases caused by RA alone (Fig. 7A). These results are consistent with previous reports of the upregulation of Raf and its activation by RA and the ability of Raf to drive differentiation [18–22]. This may reflect an ability of VAF347 in conjunction with AhR to regulate conformation of the signalsome to enable the better signaling without necessarily altering stoichiometry. It may also be a reflection of the previously reported [22] RA-induced sustained activation of the signalsome that results in driving activated c-Raf to the nucleus; i.e., thereby redirecting c-Raf from driving MEK/ERK in the cytoplasm.

Fgr and Lyn are two Src-family kinase members that have been shown to be upregulated by RA in HL-60 cells [9]. Addition of VAF347 further increased expression of Lyn and Fgr, compared to RA or VAF347 alone (Fig. 7A). VAF347 alone increased the expression of Lyn and Fgr when compared to untreated control. The combined treatment with RA + VAF347 also increased the association of Fgr with CD38 and Fgr with c-Cbl (Fig. 7B). VAF347 thus enhanced RA-induced effects on signaling molecules thought to contribute to RA-induced differentiation. VAF347 plus RA caused an association of AhR with Lyn that, although present, is at much lower barely detectable amount in untreated or RA-treated cells (Fig. 7B). This interaction is different from the aforementioned in that it is prominent and only detectable when both VAF347 and RA are present, suggesting a VAF347 dependent quantal, vs. incremental, conformational change in the signalsome.

Expression of c-Cbl was upregulated by VAF347, and VAF347 augmented the RA induced increase (Fig. 7A). This is consistent with the previously reported significance of c-Cbl expression levels to driving the RA-induced signaling that propels differentiation [15,16,23]. Immunoprecipitation showed that RA increased the interaction between c-Cbl and Fgr; and addition of VAF347 further enhanced the amount of partnered c-Cbl and Fgr (Fig. 7B). These findings are consistent with the notion that VAF347 can affect the interaction of various signalsome components, suggesting a significant overall regulatory function for AhR in the signalsome. This is furthermore corroborated by the finding that VAF347 with RA enhanced AhR interaction with c-Cbl.

Vav1 expression is up regulated by RA and is further upregulated by the addition of VAF347. By itself VAF347 also can upregulate Vav1. This is consistent with the known involvement of this guanine nucleotide exchange factor in driving myelo-monocytic differentiation. Vav1 is a requirement for RA-induced granulocytic differentiation [24]. This further supports the ability of the VAF347 AhR ligand to regulate cytosolic signaling events that propel differentiation.

**Fig. 8:**
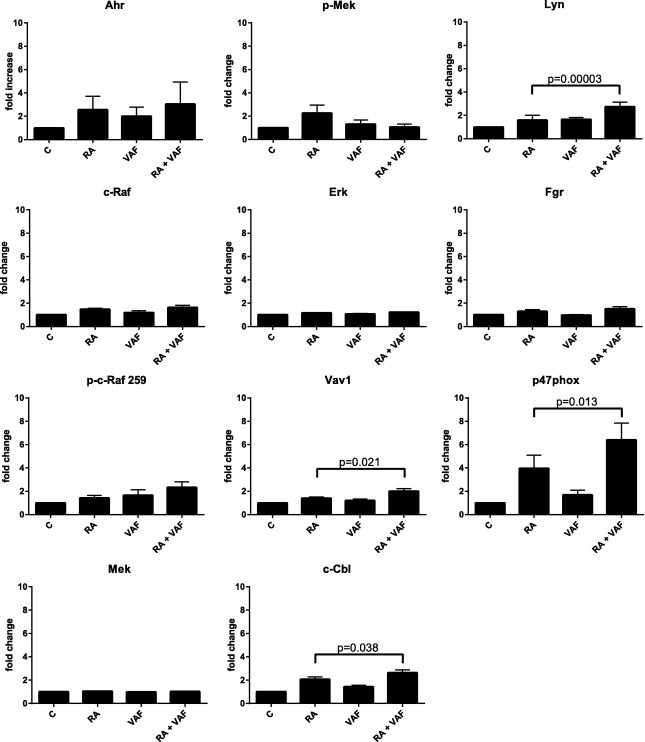
VAF347 significantly modulates selected markers of the signalsome. Western blots quantifications was performed using Versa Doc Imaging Systems, Volume Analysis Report, BioRad. Statistical analyses of all the repeats were performed using GraphPad. Means of treatment groups of interest were compared using the Paired-Samples *T* Test. Data represent the means of at least three (and no more than five) repeats ± S.E.M. A *p*-value of <0.05 was considered significant. For Lyn, Vav1, c-Cbl and p47^phox^, the statistical significance was reached (*n* = 5 repeats).

**Fig. 9:**
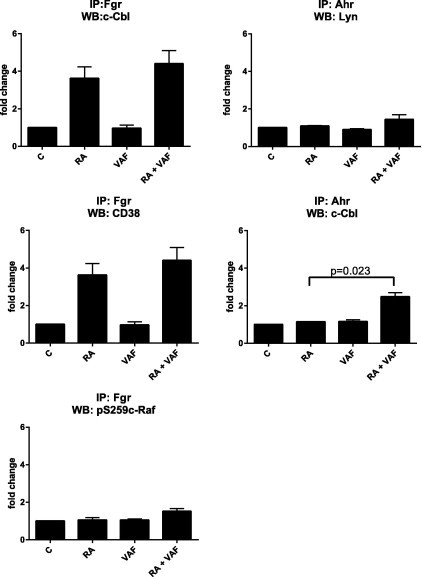
VAF347 tends to augment subcomponents of the signalsome. Western blots quantifications of the proteins associated (directly or indirectly) with either Fgr or AhR antibodies were performed using Versa Doc Imaging Systems, Volume Analysis Report, BioRad. Statistical analyses of all the repeats were performed using GraphPad. Means of treatment groups of interest were compared using the Paired-Samples *T* Test. Data represent the means of four repeats ± S.E.M. A *p*-value of <0.05 was considered significant.

VAF347 also upregulates the NADPH oxidase subunit of the respiratory burst complex, p47^phox^, in the presence of RA, but only very modestly as a single agent (Fig. 7A). Likewise, the expression of p47^phox^ appears to be particularly prominent in cells treated with RA plus VAF347 versus RA alone. Since the p47^phox^ gene is transcriptionally regulated by AhR, the results are consistent with VAF347 regulating AhR transcriptional activity in the nucleus. Upregulation of p47^phox^ is a characteristic of differentiated mature myeloid cells and an indicator of granulocytic differentiation, hence this finding supports the cytological (Fig. 3) and phenotypic marker (Fig. 4G) results presented earlier. While Fig. 7 presents the representative blots for western blot analysis and immunoprecipitation, Fig. 8 presents the quantification of the western blots (normalized to untreated control) and Fig. 9 presents the quantification of co-IPs. Several quantified markers present a trend of enhancement that does not reach significance, due to the high variability in signaling intensity between replicates. Of significance, the rank between treatment groups is maintained across replicates.

In conclusion, the present study demonstrates that VAF347 enhances RA-induced differentiation of myeloid leukemia cells toward the granulocytic lineage, and suppresses monocytic differentiation. VAF347 enhanced signalsome activation, reflected in expression, activation and interaction of its components; and enhanced expression of cell surface marker CD11b, G0/G1 cell cycle arrest, and respiratory burst. VAF347 appears to enhance several signaling events seminal to RA-induced differentiation and enhance the associated myeloid differentiation.

Taken together our results show that VAF347 enhances RA-induced granulocytic differentiation and provide novel mechanistic insights that might be exploitable for novel therapeutic modalities. The indication that AhR has a cytosolic signaling regulatory function expands the classical paradigm of AhR as a nuclear transcription factor. Combinatorial regimens incorporating VAF347 with RA may enhance the therapeutic efficacy of RA and expand its chemotherapeutic efficacy to diseases that are currently refractory to RA.

## 3 Materials and methods

### 3.1 Cell culture and treatment

Stocks of HL-60 cells were grown in RPMI 1640 media (Invitrogen, Carlsbad, CA) containing 5% heat inactivated FBS (Hyclone, Logan, UT) and 1% of an antibiotic/antimycotic agent (Invitrogen, Carlsbad, CA) in a 5% CO_2_ humidified atmosphere at 37 °C. Cell growth and viability were determined by hemocytometer counts using 0.2% trypan blue (Invitrogen, Carlsbad, CA) exclusion to assess cell viability. Cultures were initiated at a density of 0.2 × 10^6^ cells/ml and subcultured to sustain densities not exceeding 2.0 × 10^6^ cells/ml.

For experimental cultures, two million HL-60 cells were derived from a stock culture that never exceeded 1.2 × 10^6^ cells/ml and recultured in 10 ml fresh media. The cells were subsequently exposed to retinoic acid (Sigma, St. Louis, MO, USA) or 1,25-dihydroxyvitamin D_3_ (D_3_, Cayman, Mich., USA) dissolved in 100% ethanol with a stock concentration of 5 mM and 1 mM, and used at a final concentration of 1 μM and 0.5 μM, respectively, and/or (4-(3-Chloro-phenyl)-pyrimidin-2-yl)-(4-trifluoromethyl-phenyl)-amine (VAF347, EMD Millipore, Billerica, MA) at a final concentration of 20 μM. The VAF 347 was dissolved in DMSO for a stock concentration of 10 mM.

### 3.2 CD38, CD11b, and CD14 quantification

The expression of cell surface differentiation markers was quantified using flow cytometry. One million cells were centrifuged at 200 RCF for 5 min and then suspended in 200 μl PBS that contained 2.5 μl of a fluorochrome-conjugated CD38, CD14 or CD11b antibody, as indicated in Section 2. After 1 h of incubation at 37 °C with the antibodies, samples were analyzed using a BD LSRII flow cytometer (BD, San Jose, CA). Allophycocyanin (APC) was excited at 633 nm and emission was collected with a 660/20 band pass filter. Phycoerythrin (PE) was excited at 488 nm and emission was collected with a 585/42 band pass filter. Fluorescence intensity of undifferentiated control cells was compared to treated cells. Gates were set at 5% inclusion and 95% exclusion of the control population as previously reported [6,9].

### 3.3 Cell cycle quantification

One million cells were centrifuged and resuspended in 200 μl of cold propidium iodide (PI) hypotonic staining solution containing 50 μg/ml propidium iodide, 1 mg/ml sodium citrate, and 1 μl/ml Triton X-100 (all from Sigma, St. Louis, MO). Cells were incubated at room temperature for 1 h and their nuclei were analyzed by flow cytometry (BD LSRII) using 488 nm excitation. Emission was collected through a 505 long pass dichroic mirror and a 575/26 band-pass filter. Doublets were identified by a PI signal width versus area plot and excluded from the analysis, as previously described [25].

### 3.4 Respiratory burst quantification

One million cells were centrifuged at 200 RCF for 5 min. Pellets were suspended in 500 μl of PBS containing 5-(and-6)-chloromethyl-2′,7′-dichlorodihydro-fluorescein diacetate acetyl ester (DCF, Molecular Probes, Eugene, OR) with either DMSO carrier blank solution or 12-0-tetradecanoyl-phorbol-13-acetate (TPA, Sigma, St. Louis, MO) suspended in DMSO. Cells were incubated for 25 min at 37 °C and then analyzed by flow cytometry as previously described [26]. Oxidized DCF was excited by a 488 nm laser and emission was collected through a 505 long pass dichroic mirror and a 530/30 nm band pass filter. The shift in fluorescence intensity in response to TPA was used to determine the percent cells with the capability to generate inducible oxidative metabolites. Gates to determine percent positive cells were set to exclude 95% of control cells not stimulated with TPA.

### 3.5 Western blotting and Immunoprecipitation

Whole cell lysates of HL-60 cells were prepared using 200 μL of a Pierce M-PER lysis buffer solution (Pierce, Rockford, IL) containing phosphatase and protease inhibitors (Sigma, St. Louis, MO). Lysates were centrifuged at 16,000 RCF for 30 min. Protein lysates were resolved by gel electrophoresis and transferred onto Immobilon-P PVDF membrane (Millipore Corporation, Billerica MA). Equal amounts of protein lysates (25 μg) were resolved by SDS-PAGE gel electrophoresis. For immunoprecipitation, equal amounts of protein (250 μg) were precleared with 30 μL of A/G protein beads (Santa Cruz Biotechnology, Santa Cruz, CA). Lysates were then treated 1/100 volume of antibody and 50 μL of beads overnight at 4 °C prior to electrophoresis. Blots were probed with antibodies: AhR (H211), c-Cbl (C-15)- (Santa Cruz Biotechnology, Santa Cruz, CA); CD38 (BD Biosciences, San Jose, CA); phospho-p44/42 MAPK (ERK1/2) (Thr202/Tyr204) (D13.14.4E), p44/42 MAPK (ERK1/2) (137F5), pS221 MEK1/2, MEK1/2, Lyn, Fgr, Vav1, p47^phox^, c-Raf, pS259c-Raf, GAPDH (Cell Signaling, Danvers, MA). Horseradish peroxidase anti-mouse and anti-rabbit antibodies (Cell Signaling, Danvers, MA) and ECL (GE Healthcare, Pittsburgh, PA) were used during detection. Blots were repeated at least three times.

### 3.6 Wright’s-staining

HL-60 cells were treated for 96 h with combinations of RA, VAF347, and vitamin D_3_ as described above. Cytocentrifuged aliquots of control and treated cells were prepared and stained with a modified Wright’s stain using an automated stainer (Hema-Tek, Siemens, Germany). The slides were assessed for morphologic characteristics of granulocytic and monocytic differentiation. Undifferentiated blast cells were identified as cells with a high nuclear:cytoplasmic ratio, a round nucleus, prominent nucleoli, and small to moderate amounts of dark blue cytoplasm. Granulocytic or monocytic differentiation was noted if the nucleus was deeply indented or lobulated. Differentiating early granulocytic from monocytic differentiation based solely on morphology of the cells is difficult, but the cytoplasm of granulocytic cells will become pink to clear as the cell matures. Differentiated monocytic cells have a cytoplasm that is typically blue-gray, but can sometimes have fine pink granules that can give the cytoplasm a pinkish hue when viewed using a Romanowsky stain [27,28] .

### 3.7 Statistics

Statistical analyses were performed using Excel and GraphPad (GraphPad software, San Diego, CA). Means of treatment groups of interest were compared using the Paired-Samples *T* Test. Data represent the means of three repeats ± S.E.M. A *p*-value of <0.05 was considered significant.

## Authors’ contributions

RPB and AY participated in the design of the study. AY coordinated the studies. RPB and CNI carried out the assays. DMWS performed the Wright’s-staining and the morphologic assessment. CNI, RPB, DMWS and AY wrote the final manuscript.

## Conflict of interest

The authors declare no conflict of interests.

## Figures and Tables

**Fig. 6:**
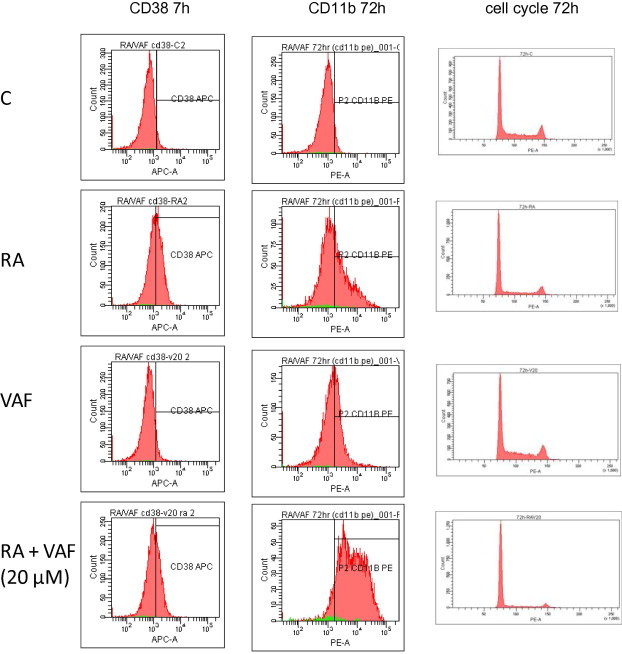
Dose response assay representative histograms. Representative histograms for CD11b PE, CD38 APC, and cell cycle for the studies quantified in Fig. 4C, D and F.
